# Seed Regeneration Potential of Canopy Gaps at Early Formation Stage in Temperate Secondary Forests, Northeast China

**DOI:** 10.1371/journal.pone.0039502

**Published:** 2012-06-22

**Authors:** Qiao-Ling Yan, Jiao-Jun Zhu, Li-Zhong Yu

**Affiliations:** 1 State Key Laboratory of Forest and Soil Ecology, Institute of Applied Ecology, Chinese Academy of Sciences, Shenyang, China; 2 Qingyuan Experimental Station of Forest Ecology, Chinese Academy of Sciences, Shenyang, China; The Pennsylvania State University, United States of America

## Abstract

Promoting the seed regeneration potential of secondary forests undergoing gap disturbances is an important approach for achieving forest restoration and sustainable management. Seedling recruitment from seed banks strongly determines the seed regeneration potential, but the process is poorly understood in the gaps of secondary forests. The objectives of the present study were to evaluate the effects of gap size, seed availability, and environmental conditions on the seed regeneration potential in temperate secondary forests. It was found that gap formation could favor the invasion of more varieties of species in seed banks, but it also could speed up the turnover rate of seed banks leading to lower seed densities. Seeds of the dominant species, *Fraxinus rhynchophylla*, were transient in soil and there was a minor and discontinuous contribution of the seed bank to its seedling emergence. For *Quercus mongolica*, emerging seedling number was positively correlated with seed density in gaps (*R* = 0.32, *P*<0.01), especially in medium and small gaps (<500 m^2^). Furthermore, under canopies, there was a positive correlation between seedling number and seed density of *Acer mono* (*R* = 0.43, *P*<0.01). Gap formation could promote seedling emergence of two gap-dependent species (i.e., *Q. mongolica* and *A. mono*), but the contribution of seed banks to seedlings was below 10% after gap creation. Soil moisture and temperature were the restrictive factors controlling the seedling emergence from seeds in gaps and under canopies, respectively. Thus, the regeneration potential from seed banks is limited after gap formation.

## Introduction

In the succession of forest ecosystems, disturbances are universal, inherent, and inevitable for maintaining the development and structure of forests [1]. Due to originating from natural regeneration after the destructive disturbances of original forest vegetation by human beings or by naturally extreme conditions, secondary forests have become major forest resources in China, accounting for more than 50% of the national total number of forests [2]–[3]. Furthermore, existing secondary forests are experiencing different types of disturbances and exhibiting obvious vegetation degradation (e.g., reduced biodiversity, decreased productivity) [4]. Promoting the natural regeneration of secondary forests undergoing various disturbances is an important approach for achieving restoration and sustainable management of secondary forests [5]–[6].

Gap disturbances, generally originating from single or multiple falling trees, are considered to be small-scale disturbances but they play a crucial role in influencing forest cycles [7]–[8] and in driving stand dynamics [4], [9]. The existing secondary forests are also experiencing gap disturbances [10]–[11]. Forest regeneration is most likely influenced by gap formation [12].

Seed regeneration (i.e., sexual reproduction) is one of the most important ways to achieve natural recovery of forests after gap disturbances [13]. Generally, successful seed regeneration must rely on adequate and viable seed sources, complete soil seed banks, and seedling and sapling banks forming from seed germination [7], [14]. According to site disturbance history, forest regeneration depends on different reproduction strategies (i.e., seed banks, seedling banks, sapling banks, and advanced regeneration) [15]. However, most previous research has focused on the seedling [7], [16] and sapling phases [17]–[18], with very few studies concerning the regeneration phase of soil seed banks and seedling emergence in gaps [19]–[20].

Seedling recruitment from the soil seed bank greatly determines the seed regeneration potential and drives the recovery direction of secondary forests [21]. The seed availability in soil is generally considered to be the bottleneck to successful seedling recruitment [22]. Furthermore, as the potential vegetation and the foundation of natural vegetation regeneration, seeds in soil play prominent ecological and evolutionary roles in linking past, present, and future plant populations and community structure and dynamics [23]–[24]. In particular, temporal variations in soil seed banks are critical for fully understanding vegetation dynamics and mechanisms [24]–[25].

There are seasonal fluctuations in the composition and abundance of soil seed banks, resulting from seed losses to deep burial, predation, death, seed decay and germination, and from seed gains through primary and secondary dispersal [26]. Following seed bank dynamics across seasons, the persistence of the seed bank can be accurately determined. It has been proven that a persistent soil seed bank (i.e., seeds in the soil that are at least one year old) has the potential to continuously supply seed sources for regeneration in disturbed ecosystems [23], [27]. The “seed bank strategy” (especially long-lived seeds in the soil) is closely associated with pioneer species, enabling quick occupation of favorable sites after an unpredictable disturbance [28]–[29]. Furthermore, the losses and gains of the seed banks mentioned above are closely related to different environmental conditions (e.g., wind regime, light, and soil temperature and moisture) [23], [30], shaped by the patterns of disturbance and stress in secondary forests [31]–[32]. After the formation of forest gaps, many changes in environmental variables will take place, with the most direct effects on the light environments [33]. Consequently, the moisture and temperature will also change with the variation in light [34]. Moreover, large variations in environments generally occur among different sizes of gaps [33]. It has been reported that in a tropical forest, the seed density of pioneer species increases with rising gap size and reaches maximal level in large gaps during early-stage formation [35]. For temperate secondary forests, however, very little work has addressed the relationship between seasonal variation in seed banks and gap formation and gap sizes.

The processes of seedling emergence from seeds and early seedling development, the key components of regeneration, are most sensitive to environmental conditions (e.g., light quantity, temperature, and moisture conditions) [36]–[37] and seed availability at a specific point [38]. The heterogeneous environmental conditions induced by gap formation or various gap sizes may alter these processes directly or change them indirectly by varying seed availability. In subtropical and tropical forests, a great deal of research has explored the relationships between seedling recruitment and environmental variation and changing seed banks undergoing gap-forming disturbances [12], [39]–[40]. However, little is known about these relationships in gaps at early developmental stages in temperate secondary forests. Furthermore, it is known that there is an obvious edge effect for species diversity of adult plants in canopy gaps [41]. The diversity and density of emerging seedlings plays a critical role in determining the direction of succession and restoration of secondary forests undergoing gap disturbances, but they are rarely considered in the gaps and along the edges of forests. Thus, for the purpose of recovering vegetation in gaps of temperate secondary forests, it is critical to understand the spatial distribution of seedlings emerging from seeds in gaps.

In this study, the seasonal dynamics of soil seed banks, the spatial distribution of seedling banks from seeds, and the relationships between seedling emergence and soil seed banks and physical environmental factors were studied in ten self-contained canopy gaps of various sizes at the early formation stage (4 years after gaps were artificially created) in temperate secondary forests in China. The main objectives were to examine: (1) whether the turnover rate of soil seed banks varies with gap formation; (2) how the spatial patterns of seedling banks respond to the creation of canopy gaps of different sizes; and (3) how seedling emergence correlates with the seed availability in the soil and environmental factors at the early formation stage of gaps in temperate secondary forests.

## Methods

### Study Site Description

The study was conducted at the Qingyuan Experimental Station of Forest Ecology (QESFE) of the Institute of Applied Ecology, Chinese Academy of Sciences, in Liaoning Province, Northeast China (124°54′E, 41°51′N, 500–1100 m a.s.l.). The climate is a continental monsoon type with a strong monsoon windy spring, a warm and humid summer, and a dry and cold winter. The mean annual air temperature is 4.7°C, with the minimum of −37.6°C in January and the maximum of 36.5°C in July. Annual precipitation ranges from 700 to 850 mm, of which 80% falls in June, July and August. The growing season is from early April to late October [11].

The study site at QESFE was originally covered with mixed broadleaved-Korean pine forests until the 1930s and subsequently subjected to decades of unregulated timber removal. The original forests were completely cleared after a large fire occurred in the early 1950s, and the site was replaced by secondary forest stands [2]. The representative selected secondary forest stands at QESFE were a mixture of naturally regenerating broadleaved native tree species, mainly dominated by *Fraxinus rhynchophylla* Hance, *Acer mono* Maxim., and *Quercus mongolica* Fisch., etc. [42]. At the end of 2004 (winter), by cutting and removing all vegetation higher than 2 m in secondary forests, ten approximately elliptical gaps of various sizes with a length to width ratio of 3∶2 were created, including two large gaps of >500 m^2^ (LGs), three medium gaps of 150–500 m^2^ (MGs), and five small gaps of <150 m^2^ (SGs) in size. These gaps were approximately 20 meters apart and located on the same south-north slope aspect and mid-slope position. In addition, with the same history of forest management, the ten artificial gaps shared similar soil type, topography (including altitude and slope), and vegetation composition. Simultaneously, using the 16 equiangular elliptic sectors (16EES) method [43]–[44], the sizes of the artificial canopy gaps were precisely calculated. The diameter at breast height (DBH) and height of gap border trees were measured using the Laser Ranging System (LI-RD1000, Laser Technology, Inc. USA). The site characteristics of the canopy gaps in this study are summarized as shown in Table 1. There were two reasons for using a small number of large gaps in the present study: first, it was very difficult to find suitable sites for creating large gaps without complicating the topographical conditions and vegetation types, and secondly, removal of canopy trees in natural forests has been highly regulated by governmental policy under China’s Natural Forest Protection Project.

**Table 1:** General description of the forest gaps investigated in the study of Chinese temperate secondary forests.

									Canopy openness (%)	
Forest gap	Area (m^2^)	Altitude (m)	Slope (°)	Height of gap border trees (m)	DBH of gap border trees (cm)	Vegetation composition of gap border trees (%)	Quadrats along the south-north direction of gap	Quadrats along the east-west direction of gap	In gap	Under canopy
G1	698.4	760	16	9.53±0.65	11.26±1.04	*Tilia tuan* (23), *Fraxinus rhynchophylla* (21), *Acer mono* (16)	12	10	25	15
G2	587.3	754	15	12.95±0.65	15.45±0.98	*Fraxinus rhynchophylla* (39), *Quercus mongolica* (18), *Populus davidiana* (14)	10	8	24	16
G3	356.2	736	15	16.19±1.09	22.15±2.30	*Acer mono* (35), *Quercus mongolica* (35), *Juglans mandshurica* (10)	8	8	20	18
G4	345.9	768	17	15.33±1.26	19.22±2.01	*Quercus mongolica* (56), *Fraxinus rhynchophylla* (21), *Tilia tuan* (21)	8	8	17	15
G5	196.8	721	17	9.68±0.82	10.83±1.33	*Acer mono* (37), *Fraxinus rhynchophylla* (30), *Quercus mongolica* (21)	8	8	19	16
G6	128.8	775	18	10.75±0.60	11.36±1.05	*Acer mono* (81)	8	8	16	13
G7	109.6	756	19	9.11±1.54	14.31±3.95	*Acer mono* (44%), *Tilia tuan* (22), *Ulmus laciniata* (22)	6	6	15	11
G8	84.2	728	16	9.59±0.88	9.15±2.08	*Fraxinus rhynchophylla* (65), *Acer mono* (24)	6	6	15	12
G9	74.7	755	16	11.25±1.05	16.60±2.99	*Acer mono* (67), *Ulmus laciniata* (25)	6	6	15	13
G10	52.3	771	20	8.55±1.11	11.71±3.10	*Quercus mongolica* (36), *Acer mono* (36), *Ulmus laciniata* (18)	6	6	14	11

DBH =  mean tree diameter at breast height (±S.E.). From Gap 1 to Gap 10, gap size gradually decreased.

### Sampling Points Setting

In field investigations, we considered each canopy gap as a self-contained unit [20]. Our preliminary experiment indicated that the data from soil seed banks collected from two transects running along the longitudinal and latitudinal axes in each elliptical gap were sufficient to represent the characteristics of a spatial distribution of seed banks [11]. Therefore, in this study we opted to investigate only two transects in practical operation: one longitudinal transect ran along the south-north direction of the canopy gap (in the direction from lower to upper slope) and the other latitudinal transect ran along the east-west direction of the gap (parallel to slope surface) [11]. Sampling points were set 3 m apart along each transect from the gap center to the canopies adjacent to the gaps on all four sides. In total, data were collected on 12 sampling points for the smallest gap, 22 points for the largest gap, and 152 points for all ten canopy gaps (Table 1). At each sampling point, the soil seed bank, seedling emergence, and environmental conditions were monitored during 2008–2009. These investigations were considered to have been conducted during the early formation stage (i.e., 4–5 years) of gaps after they were artificially created.

### Soil Seed Bank Sampling

Samples were collected to involve three crucial periods in the seasonal dynamics of soil seed banks in temperate forests: (1) at the end of most of the seed fall into the seed bank (in November 2008, “autumn sample”), (2) after seed dispersal and before the beginning of the spring germination event (in April 2009, “spring sample”), and (3) when most seeds had already emerged but prior to the current-year seed shedding (in August 2009, “summer sample”). For each sampling period, soil collection was repeated at almost the same points, avoiding the effects of the previous samplings. Prior to investigation of the soil seed banks, seeds were collected from living plants in 2007 to identify the species in the seed banks [11].

Aiming to reduce the variations in seed distribution resulting from local spatial heterogeneity, five soil samples around each sampling point were taken and combined to form a composite sample for each sampling period. The soil samples of 0–5 cm were collected using a hollow steel cylinder 70 mm in diameter. Seed extraction was conducted to assess soil seed bank composition of all species [11], [45]. According to our observations, the sizes (i.e., length, width, and height) of seeds for the dominant tree species were bigger than a mesh size of 0.5 mm in the present study (data not shown). Therefore, after air-drying, the soil samples with seeds were ground softly by hand to avoid damaging the seeds, and only soil particles smaller than 0.5 mm passed through the sieve [11]. After sieving, the seeds were extracted from the rest of soil particles bigger than 0.5 mm by hand and subsequently identified. Then, seed viability was tested using the “tetrazolium dyeing method” [46] and only viable seeds (including both germinable and dormant seeds) were included in the present study.

### Seedling Emergence Monitoring

Seedling emergence quadrats (1 m×1 m) were set to correspond to sampling points for seed bank collection along each transect. Seedlings of dominant gap tree species that originated from seeds in each quadrat were recorded, counted, and removed at 15-day intervals during the growing season (from early April to late October) of 2009.

### Environmental Condition Monitoring

Environmental conditions (including light availability, soil moisture, and temperature) were monitored every 15 days during the growing season of 2009.

Light availability: Defined as photosynthetically active radiance (PAR), light availability at each sampling point was monitored automatically using a LI-1400 system (LI-190, Li-COR, Nebraska, USA) with a quantum logger (Watch Dog data logger, QMSW-SS, USA). PAR was represented as the Photosynthetic Photon Flux Density (PPFD) (µmol·m^−2^·s^−1^) in the 400–700 nm waveband. Horizontal readings (sensor head held at 0°) were set on the plate at the height of 1.3 m above the forest floor at each point. PPFD was logged every 1 s, and the 10-min average was stored in a data logger (21x, Campbell Scientific, Utah, USA). The observations were conducted at 15-day intervals under two different weather conditions (i.e., sunny days and overcast days). Furthermore, the observations lasted continuously between 7:00–17:00 (GMT+8:00) for each observation day [33].

Soil water content: The time domain reflectometry (TDR) (TRIME-HD, Germany) was used to estimate the soil water content in a 15-cm soil profile (*SWC*_15_). TDR probes with two parallel steel rods (diameter, 6 mm; length, 15 cm) were inserted into the soil at 45° angles from the undisturbed litter layer. There were three replicates at each sampling point for each measurement.

Soil temperature: The soil temperature at 5 cm depth (*T*_5_) was measured by a thermometer (TM-150, Custom, Tokyo, Japan) at each sampling point along two transects. For each measuring day between 7:00–17:00, the soil temperature data were collected at hourly intervals. Only the mean (*T*_mean_), maximum (*T*_max_), and minimum (*T*_min_) soil temperatures were used in the analyses.

### Data Analysis

Data for seeds were converted to the number of viable seeds m^−2^ (i.e., seed density). Seed bank diversity of all species collected at each sampling point was estimated using the Shannon-Wiener index:
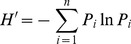
(1)where
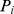
was the proportional abundance of the
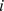
th species [47].

The Sokal and Sneath similarity indices [48] were calculated for species compositions of all species collected in seed banks between November 2008 and April 2009 (autumn-spring interval), between April 2009 and August 2009 (spring-summer interval), and between November 2008 and August 2009 (autumn-summer interval) respectively, in gaps and under canopies.

Using normal probability plots, all data were tested for normality and transformed when appropriate. A mixed-model repeated measures analysis of variance (RMANOVA) was used to test the effects of gap size on seed density, diversity, and similarity of seed banks at the sampling points across the three time intervals. In the RMANOVA, gap size was treated as a random effect, and time was treated as a fixed effect [49] (SPSS software, 16th edition, Chicago, USA). When the interactions between the gap size and sampling season were significant, one-way ANOVA and Tukey’s HSD post-hoc tests were applied (SPSS software, 16th edition, Chicago, USA) to test the effects of gap size on density, diversity, and similarity of seed banks by sampling season and the effects of sampling season on seed bank information by gap size. Differences were considered significant at *P*<0.05.

The relationship between the number of year-round emerging seedlings and their corresponding seed densities in soil seed banks in April 2009 was tested at the sampling points in gaps and under canopies using Pearson’s bivariate correlations (SPSS software, 16th edition, Chicago, USA).

The relationships between the selected environmental variables (light availability, soil temperature, and soil water content) and emerging seedling densities were analyzed with Canonical Correspondence Analysis (CCA) among selected gaps and canopies using CANOCO 4.0 [50]. CCA is particularly useful for analyzing species-environment relationships [51]. Prior to analysis, the seedling species data were log transformed. To examine the significance of the canonical axis of the partial CCA, a permutation test was conducted with 199 permutations under a reduced model. The results were displayed as a CCA bi-plot of species and environmental variables.

An unpaired, 2-tailed t-test (SPSS software, 16th edition, Chicago, USA) was used to distinguish seedling densities of dominant species between the gaps and canopies adjacent to the gaps. In the analysis of the spatial distribution of emerging seedlings, seedling density at a certain position from the gap center was represented by the mean value of seedling densities from two transects at the same distance from the center. One-way ANOVA and Tukey’s HSD post-hoc tests were applied (SPSS software, 16th edition, Chicago, USA) to distinguish seedling densities among different positions from the canopy near the gap to the center of the gap. Differences obtained at a level of *P*<0.05 were considered significant.

## Results

### Seasonal Dynamics of Soil Seed Banks

**Figure 1:**
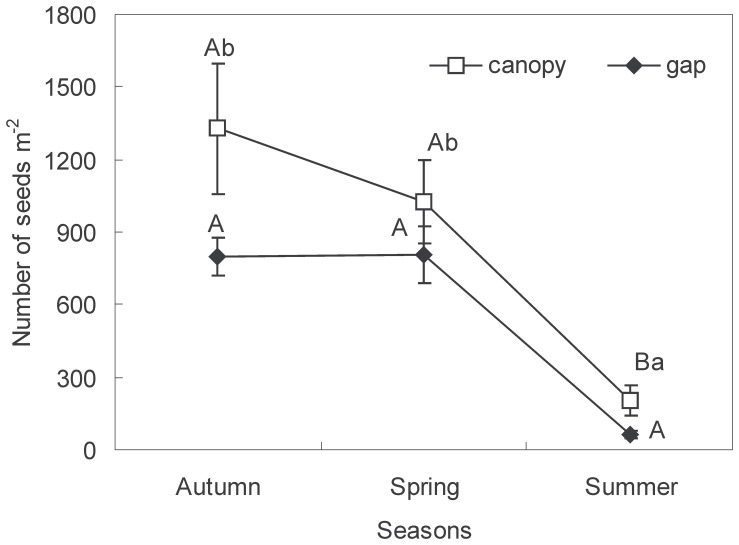
Seasonal dynamics of total soil seed banks in gaps and under canopies in the study on the role of seed banks in the regeneration potential of Chinese temperate secondary forests. Different lower case letters indicate significant seasonal differences in seed density in both the gaps and the canopies (*P*<0.05). The values of seed density with the same upper case letters do not differ significantly between the gaps and the canopies at the same time (*P*<0.05). Error bars indicate means ± S.E.

Season had a significant effect on the average seed density both in gaps (*F*_2,10_ = 21.22, *P*<0.01) and under canopies (*F*_2,10_ = 10.49, *P*<0.05). Furthermore, in both gaps and canopies, the total number of seeds decreased gradually from autumn to the summer of the next year, and the seed density in summer was significantly lower than that in the other two seasons (Fig. 1). However, none of the two-way interactions between gap size and season was significant with respect to seed density (*P*>0.05). During all three sampling periods, seed densities under canopies were higher than those in the gaps and especially there was a significant difference in summer (*P*<0.05) (Fig. 1).

**Figure 2:**
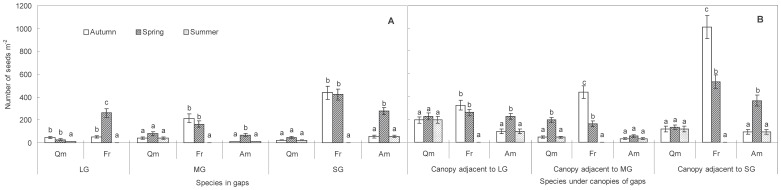
Seasonal dynamics of soil seed banks for three species in gaps (A) and under canopies (B) in the study on the role of seed banks in the regeneration potential of Chinese temperate secondary forests. Different lower case letters indicate significant seasonal differences in seed density for the same species in the gaps or under the canopies (*P*<0.05). Error bars indicate means ± S.E. Qm = *Quercus mongolica*; Fr = *Fraxinus rhynchophylla*; Am =  *Acer mono*; LG = Large gaps; MG = Medium gaps; SG = Small gaps.

Seasonal dynamics of the individual species in the seed banks were inconsistent with that of the overall seeds mentioned above. In large gaps, only seeds of *Q. mongolica* and *F. rhynchophylla* appeared, and the seed density of *F. rhynchophylla* significantly increased from autumn to spring of the next year (*P*<0.05; Fig. 2A). Furthermore, the same tendency for variation was true of *A. mono* in the MGs, SGs, and under the canopy of the LGs and SGs, and true of *Q. mongolica* under the canopy of the MGs (*P*<0.05; Fig. 2). *F. rhynchophylla* did not appear in the summer either in the gaps or under the canopies of gaps (Fig. 2). Generally, the seed densities of specific species under the canopy were higher than that in the corresponding gap.

**Figure 3:**
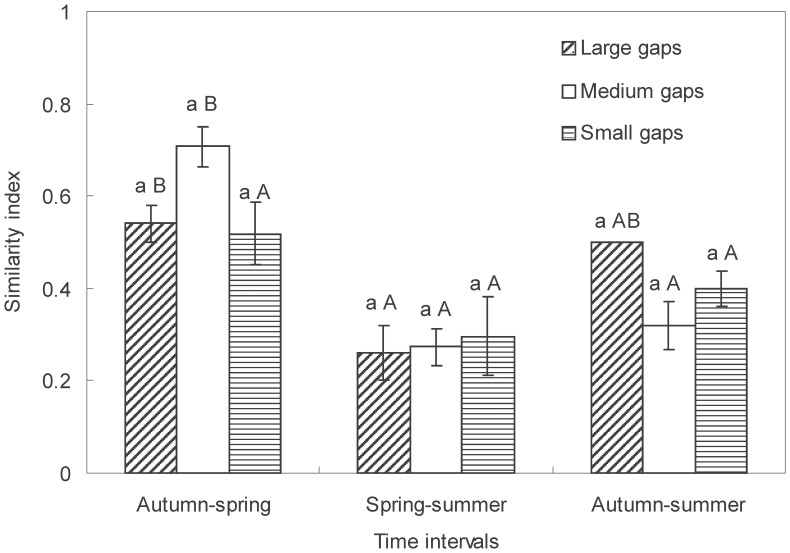
Similarities in species composition of soil seed banks at three time intervals (autumn-spring, spring-summer, and autumn-summer) in forest gaps of three different sizes. Different lower case letters indicate significant differences in similarities of species composition in different sizes of gaps at the same interval (*P*<0.05). The values for seed bank similarity with the same upper case letters do not differ significantly among the three time intervals in the same gap size (*P*<0.05). Error bars indicate means ± S.E.

In forest gaps, both seasons (*F*_2,10_ = 92.80, *P*<0.0001) and the two-way interaction between seasons and gap sizes (*F*_2,10_ = 16.03, *P*<0.01) had significant effects on the similarity for the species composition of soil seed banks. At the same time interval, there were no significant effects of gap size on the similarity of species composition in soil seed banks (*P*>0.05) (Fig. 3). In large gaps, after most of the seeds had emerged (i.e., spring-summer interval), similarity of the species composition of seeds was significantly lower than that at the autumn-spring interval (*P*<0.05). In medium gaps, after most of the seeds had arrived in the seed bank and before the beginning of spring germination event (i.e., autumn-spring interval), similarity of species composition in the soil seed bank reached its highest (*P*<0.05). For small gaps, there was no significant effect of season on seed composition similarity (*P*>0.05). However, for canopies adjacent to the gaps, only season (*F*_2,10_ = 2.00, *P*<0.05) significantly affected similarity of species composition in seed banks. Additionally, the similarity of species composition in soil seed banks between the gaps and the canopies adjacent to the gaps fluctuated across seasons, from 0.75 in the autumn of 2008 to 0.80 in the spring of 2009 and to 0.55 in the summer of 2009 (data not shown).

**Figure 4:**
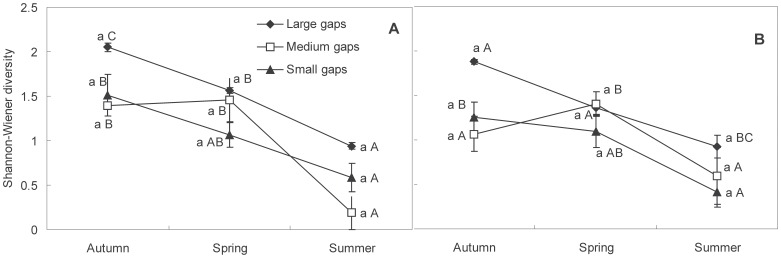
Shannon-Wiener diversity of soil seed banks in three different sizes of forest gaps (A) and canopies adjacent to the gaps (B) across seasons. The values of seed bank diversity with the same lower case letters do not differ significantly among different sizes of gaps in the same season (*P*<0.05). Different upper case letters indicate significant differences in the seed bank diversity of species across seasons in the same size of gaps (*P*<0.05). Error bars indicate means ± S.E.

For the gaps in the present study, both season (*F*_2,10_ = 317.30, *P*<0.0001) and the two-way interaction between season and gap size (*F*_2,10_ = 26.92, *P*<0.01) influenced the Shannon-Wiener diversity of the soil seed bank. In the same season, however, there were no significant effects of gap size on seed bank diversity of species composition in both the gaps and the canopies adjacent to the gaps (*P*>0.05) (Fig. 4). Overall, the diversity of the soil seed bank in the gaps gradually decreased from the autumn of 2008 to the summer of 2009 (*P*<0.05) (Fig. 4A). Furthermore, the seed bank diversity for canopies adjacent to the gaps varied significantly across seasons (*F*_2,10_ = 6.40, *P*<0.05) and across the interaction between season and gap size (*F*_2,10_ = 6.55, *P*<0.05). Under canopies adjacent to large and small gaps, seed bank diversity in autumn was significantly higher than that in the other two periods (*P*<0.05) (Fig. 4B). However, seed diversity under canopies adjacent to medium gaps was the highest in the spring of 2009 (*P*<0.05) (Fig. 4B). Furthermore, the seed diversity in the gaps was slightly higher than that under the canopies for the three sampling periods (*P*>0.05) (data not shown).

### Spatial Pattern of Emerging Seedlings

Only three types of dominant tree species (*F. rhynchophylla*, *Q. mongolica*, and *A. mono*) were observed in the gaps and under the canopies during the study period. Regardless of gap size, seedling density of *F. rhynchophylla* under canopies (31±15 seedlings m^−2^) was almost two times higher than that in the gaps (16±2 seedlings m^−2^) (*F*_133_ = 13.89, *P*<0.05). Conversely, seedling densities of *Q. mongolica* (4±0.6 seedlings m^−2^) and *A. mono* (2±0.3 seedlings m^−2^) in the gaps were almost two times higher than those under the canopies, respectively (*P*>0.05).

**Figure 5:**
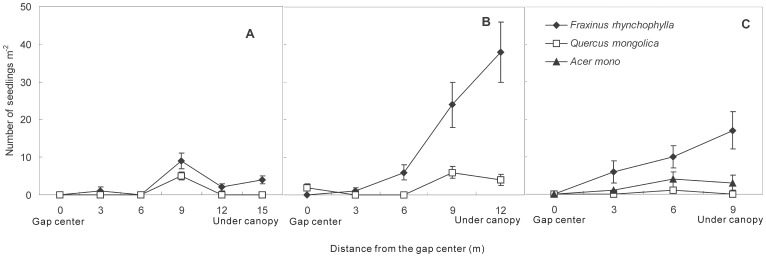
Spatial distribution of newly emerged seedlings from seeds in soil at various positions from the gap center in large gaps (A), medium gaps (B), and small gaps (C). Error bars indicate means ± S.E.

In large gaps, only seedlings of *F. rhynchophylla* and *Q. mongolica* emerged during the growing season (Fig. 5A). From the gap center to the forest canopy, the seedling densities fluctuated and reached the highest (9±2 seedlings m^−2^ for *F. rhynchophylla*, and 5±1 seedlings m^−2^ for *Q. mongolica*) near the edge of the gaps (i.e., 9 m away from the gap center) (*P*<0.05).

Seedlings of *F. rhynchophylla* and *Q. mongolica* also occurred in medium gaps across the entire growing season (Fig. 5B). For *F. rhynchophylla*, seedling density significantly increased from the gap center to the canopy (*P*<0.05) and finally reached its highest under the canopies (38±5 seedlings m^−2^) in the MGs. Spatially, seedling density of *Q. mongolica* fluctuated and reached the highest (6±1 seedlings m^−2^) at the edge of the gaps (i.e., 9 m away from the gap center) (*P*<0.05).

In small gaps, there were three types of seedlings (i.e., *F. rhynchophylla*, *Q. mongolica*, and *A. mono*) that appeared throughout the growing season (Fig. 5C). The seedling density of *F. rhynchophylla* significantly increased from the gap center to the canopy (*P*<0.05) and was the highest under the canopies (17±2 seedlings m^−2^). For the other two species, seedling densities fluctuated spatially over the entire growing season.

### Relationship Between Emerging Seedlings and Soil Seed Banks

**Figure 6:**
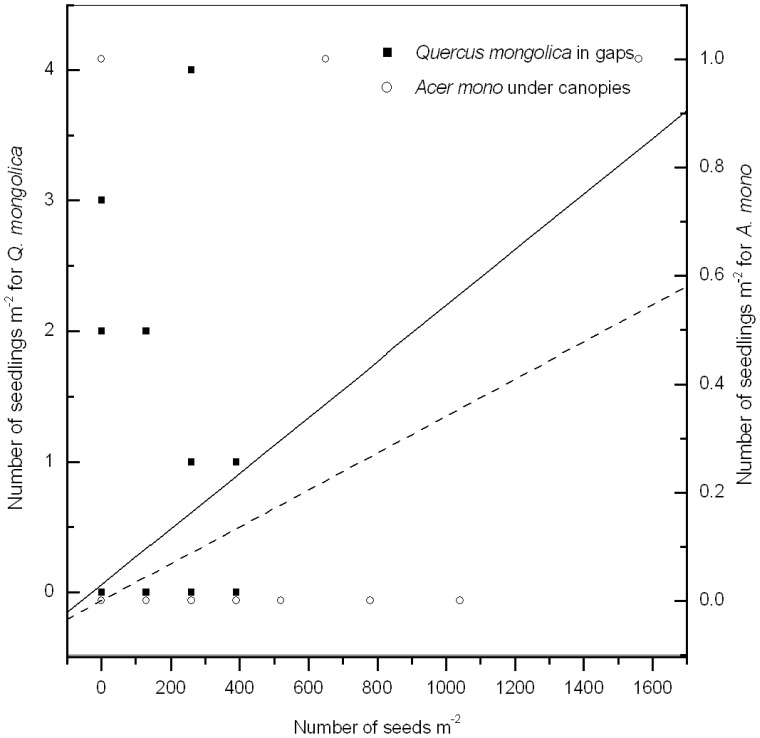
Relationship between emerging seedling density and seed density in April 2009 for *Quercus mongolica* in gaps and *Acer mono* under canopies.

There were only three types of seedlings (i.e., *F. rhynchophylla*, *Q. mongolica*, and *A. mono*) that emerged from seeds during the study period. Thus, the correlations between these emerging seedlings and their corresponding seed banks in April 2009 were analyzed among the selected gaps. For *F. rhynchophylla*, the correlation was not significant either in the gaps or under the canopies of various gap sizes (*P*>0.05). However, emerging seedlings of *Q. mongolica* were positively correlated with its soil seed bank in the forest gaps (*R* = 0.32, *P*<0.01, *n* = 99; Y*_Q. mongolica_* = 0.06+0.01X*_Q. mongolica_*) (Fig. 6), especially in the gaps of medium (*R* = 0.42, *P*<0.01, *n* = 39) and small sizes (*R* = 0.58, *P*<0.0001, *n* = 45). Furthermore, there was a significantly positive correlation between the seedlings of *A. mono* and its seed bank just under canopies (*R* = 0.43, *P*<0.01, *n* = 36; Y*_A. mono_* = 0.04+0.03X*_A. mono_*) (Fig. 6).

### Relationship between Seedling Emergence and Environmental Factors

**Figure 7:**
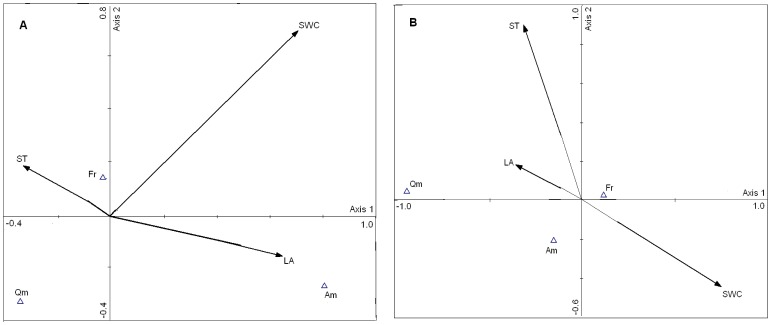
Canonical Correspondence Analysis (CCA) biplot ordination of seedling density for three plant species (Δ) and environmental variables (arrows) in gaps (A) and under canopies (B). Species abbreviations: Am = *Acer mono*; Qm = *Quercus mongolica*; Fr = *Fraxinus rhynchophylla*. Environmental variable abbreviations: ST = soil temperature; LA = light availability; SWC = soil water content.

The relationships between the seedling density of plant species and three environmental variables (i.e., soil temperature, light availability, and soil water content) in both gaps and canopies were expressed using the CCA ordination diagram (Fig. 7). For gaps in the present study, the first axis explained 8.9% of the variation and was most strongly correlated with soil water content (SWC, 0.705) and light availability (LA, 0.646). These data indicated that from left to right on the first axis, SWC and LA gradually increased. The second axis explained 4.2% of the variation and was most strongly correlated with SWC (0.696; Table 2), indicating that SWC gradually increased from the bottom to the top of the second axis. The effects of the environmental variables on the distribution of seedling emergence in the gaps were ranked as: SWC > LA > soil temperature (ST). With an increase in ST, seedlings of both *F. rhynchophylla* and *Q. mongolica* gradually increased. Seedlings of *A. mono* tended to occur in places of greater light availability found in the gaps (Fig. 7A).

**Table 2:** Correlations between Canonical Correspondence Analysis (CCA) ordination axes and factors both in gaps and under canopies in the study on the role of seed banks in the regeneration potential of Chinese temperate secondary forests.

	In gaps				Under canopies			
	Axis 1		Axis 2		Axis 1		Axis 2	
Factors	Correlation	*P*	Correlation	*P*	Correlation	*P*	Correlation	*P*
Light availability	0.646	0.004	−0.158	0.317	−0.361	0.071	0.179	0.247
Soil temperature	−0.335	0.078	0.184	0.269	−0.319	0.091	0.895	0.000
Soil water content	0.705	0.000	0.696	0.003	0.748	0.000	−0.444	0.048

For canopies adjacent to the gaps, the first axis explained 15.5% of the variation and was most strongly correlated with SWC (0.748). The second axis explained 0.6% of the variation and was most strongly correlated with ST (0.895) and SWC (−0.444; Table 2). The effects of the environmental variables on the distribution of seedling emergence were ranked as: ST > SWC > LA. With an increase in LA, seedling densities for both *F. rhynchophylla* and *A. mono* tended to decline. Seedlings of *Q. mongolica* tended to occur in places of greater light availability under the canopies (Fig. 7B).

## Discussion

Soil seed banks have various density and diversity responses to gap formation in tropical forests [52]. The same condition also occurred for the temperate canopy gaps at the early formation stage of the present study. We found that, compared with the surrounding closed stand, forest gaps could promote species diversity of seed banks at all of the sampling times, which is consistent with previous reports of temperate forests [41]. However, the seed density in soil seed banks had not improved after gap formation (Fig. 1), contrary to other studies [53]. All of these findings indicate that at the early stage of gaps in temperate secondary forests, gap formation can favor the invasion of more types of species in the seed bank. However, gap formation can also speed up the turnover rate of seed banks with lower densities.

In the present study, gap size plays little role in controlling the seasonal variations in species-composition similarity and diversity of seed banks (Fig. 3, 4). However, there is a significant effect of gap size on seed density for specific species. Our results indicated that for *Q. mongolica* in spring and summer, the highest and lowest density appeared in the MGs and LGs, respectively (Fig. 2A). However, for the autumn sampling, the seed density of *Q. mongolica* decreased with declining gap size. For *F. rhynchophylla* and *A. mono*, the highest density only existed in the SGs during the three sampling periods (Fig. 2A). As the pioneer species in temperate secondary forests [54], *Q. mongolica* generally occurs in the MGs (150–500 m^2^), which is inconsistent with pioneer species in tropical forests [35]. In our study, however, most seeds of other non-pioneer tree species appeared in the SGs (<150 m^2^). Obviously, the abundance of seeds for tree species does not increase with increasing gap size in temperate forests at early stages of formation.

According to the seasonal variation in seed banks, the persistence of seeds can be clearly discriminated [23]. In the present study, few seeds of *F. rhynchophylla* appeared during the period after seedling emergence (i.e., summer sampling period), either in the gaps or under the canopies of gaps (Fig. 2). This demonstrates that in temperate secondary forests, seeds of *F. rhynchophylla* are transient in the soil and their viability can be sustained for less than six months. Consequently, there was little and discontinuous contribution of seed banks to seedling emergence in the present study, consistent with another study on *F. rhynchophylla* seedling regeneration in temperate secondary forests [55]. On the contrary, there are persistent seed banks for the other two dominant tree species (i.e., *Q. mongolica* and *A. mono*) to continuously supply seed resources for seedling emergence (Fig. 6).

Based on the spatial distribution characteristics of the seedlings that emerged from seeds, gap formation was much more favorable for the emergence of *Q. mongolica* and *A. mono* seedlings. The percentage of emerging seedlings accounting for seed abundance was 10.3±4.7% and 0.9±0.1% for *Q. mongolica* and *A. mono*, respectively. Thus, it can be concluded that from the perspective of seed regeneration potential, both *Q. mongolica* and *A. mono* are “gap-dependent” species in temperate secondary forests. After tree-fall gap formation or at the early formation stage of gaps, these gap-dependent species will invade first due to a favorable environment having been created (e.g., more light and higher temperatures) [56]. Furthermore, the highest density of *Q. mongolica* seedlings existed at the edge of the LGs and MGs, and for *A. mono*, the highest occurred at the edge of the SGs. Obviously, there are edge effects of gaps on the seedling emergence of these gap-dependent tree species. For *F. rhynchophylla*, however, most seedlings emerging from seeds appear in the closed-canopy forests (Fig. 5), and the percentage of emerging seedlings accounting for seed abundance was 20.1±5.6%. Although these three dominant tree species are light-demanding in the seedling phases [57], gap formation plays a rather critical role in the early development of *Q. mongolica* and *A. mono* seedlings in temperate secondary forests.

Comparing gaps with closed-canopy forests, there are different restricting factors controlling the seedling emergence from seeds. After the creation of gaps, the evaporation of soil moisture increases sharply due to the increases in light and temperature [33]. However, sufficient water in the soil is rather important for maintaining seed viability [58]. Thus, soil moisture played a restricting role in seedling emergence in tree-fall gaps (Fig. 7A). Under the premise that soil moisture promotes seed germination in the gaps, seedlings tend to emerge in high temperature and light environments. In the closed-canopy forests in our study, soil temperature had a critical limiting effect on seedling emergence for the three dominant tree species (Fig. 7B). Compared with the gaps, due to the smaller canopy openness and lower light levels in the closed canopies, soil moisture is high enough to break seed dormancy. However, soil temperature may be much lower and restrict the processes of seedling emergence from seeds in temperate forests [33]. Therefore, based on the appropriate soil temperature, seedlings of *F. rhynchophylla* and *A. mono* tended to emerge in lower light conditions, whereas *Q. mongolica* seedlings tended to develop under higher light conditions. Consequently, due to the persistent attributes of the seed banks and different light demands for seedling emergence of *Q. mongolica* and *A. mono*, there were significantly positive correlations between seedlings and seed banks in forest gaps and under canopies, respectively (Fig. 6, 7). This proves that during seedling recruitment from soil seed banks, both *F. rhynchophylla* and *A. mono* are much more shade-tolerant in temperate secondary forests.

These findings suggest that the formation of gaps, to some extent, facilitates the accumulation of more types of species in seed banks but speeds up the turnover rate of the seed bank. Furthermore, gap formation can promote the emergence of *Q. mongolica* and *A. mono* seedlings and there is an edge effect of the gap on seedling emergence. After the gap is created, the contribution of seed banks to seedlings of these two gap-dependent species is below 10% under completely natural conditions. Soil moisture and temperature are the restrictive factors controlling seedling emergence in gaps and under canopies, respectively. Thus, the regeneration potential of soil seed banks is limited after gap formation.
